# Causal Inference in Radiomics: Framework, Mechanisms, and Algorithms

**DOI:** 10.3389/fnins.2022.884708

**Published:** 2022-06-20

**Authors:** Debashis Ghosh, Emily Mastej, Rajan Jain, Yoon Seong Choi

**Affiliations:** ^1^Department of Biostatistics and Informatics, Colorado School of Public Health, Aurora, CO, United States; ^2^Computational Biosciences Program, University of Colorado Anschutz Medical Campus, Aurora, CO, United States; ^3^Department of Radiology and Neurosurgery, New York University Langone Medical Center, New York, NY, United States; ^4^Department of Radiology, Yonsei University College of Medicine, Seoul, South Korea

**Keywords:** latent causal effect, link-free inference, medical imaging, personalized medicine, sufficient dimension reduction

## Abstract

The widespread use of machine learning algorithms in radiomics has led to a proliferation of flexible prognostic models for clinical outcomes. However, a limitation of these techniques is their black-box nature, which prevents the ability for increased mechanistic phenomenological understanding. In this article, we develop an inferential framework for estimating causal effects with radiomics data. A new challenge is that the exposure of interest is latent so that new estimation procedures are needed. We leverage a multivariate version of partial least squares for causal effect estimation. The methodology is illustrated with applications to two radiomics datasets, one in osteosarcoma and one in glioblastoma.

## 1. Introduction

Radiomics explores relationships between image-derived characteristics of a tumor and other parameters, including clinical outcomes and genomic profiles, including gene expression, somatic mutations, and DNA methylation (Mazurowski, 2015). In particular, several groups have built classifiers to predict tumor molecular phenotypes using radiomic inputs (e.g., Kickingereder et al., 2016; Rios Velazquez et al., 2017; Yip et al., 2017; Xi et al., 2018). More recently, there has been tremendous interest in using modern machine learning and in particular deep learning tools in order to build state-of-the-art classifiers for predictions (Lao et al., 2017; Li et al., 2017; Parnian et al., 2020).

In spite of their state-of-the-art performance, use of these complex models comes at a cost. Because many of these classifiers are “black-box” in nature, clinicians consequently have a difficult time understanding the predictions. More generally, most work in radiomics has focused on pattern-based associations in the data with a machine learning viewpoint in one of two ways. First, these analyses could take the form of clustering algorithms, such as t-SNE or UMAP, in which interesting clusterings lead to followup discoveries. Second, a classification or supervised learning framework could be adopted in which the radiomics features could be used to predict a class label or phenotype of interest. For this approach, a typical evaluation is classification accuracy of the ROC curve or F1 values where higher values are better. While there has been tremendous successes in radiomics with these machine learning techniques, it still remains elusive from the end goal of developing mechanistic insights into tumorigenesis.

In this article, we seek to introduce causal modeling concepts into radiomics. While there has been much work on using these ideas in genomics (e.g., Huang and Pan, 2016; Aung et al., 2020) and brain imaging (e.g., Chén et al., 2018), their application to radiomics has not occurred. We argue that adopting this viewpoint in radiomics has the following advantages:

1. It allows one to view radiomics as measurements of properties of the tumor and its characteristics.
2. Developing a causal model allows one to link tumorigenic mechanisms to observed data.
3. The causal inferential pathway is compatible with the trend toward systems biology (Alon, 2019) while not being as purely reductionist as approaches such as those based on mathematical models or ordinary differential equations.

However, a challenge that this approach introduces is that we must view the tumor as a latent construct, and causal inference with latent structures is much more challenging. There has been much recent interest in the use of latent class modeling of treatment effects on outcomes (Collins and Lanza, 2009). Bandeen-Roche et al. (1997) proposed a latent class modeling approach in which pseudodraws from the inferred latent class distribution are then used to fit regression models on the outcome. Multiple pseudodraws are generated, and the multiple regression results are combined using Rubin's imputation rules (Little and Rubin, 2019). A simpler approach is to use a classify/analyze approach (Clogg, 1995) in which each individual is assigned to a latent class, and then the group assignment is used as a covariate for which standard propensity score methods can be applied. In a recent study, Schuler et al. (2014) compared these approaches, along with a joint modeling approach developed by Kang and Schafer from an unpublished technical report at the Methodology Center of Penn State University. The approaches can be broadly grouped as being 1-step vs. 3-step approaches (Asparouhov and Muthén, 2014). The former methods fit a joint model describing both the latent classes as well as the latent classes' effect on the outcome. The latter go through a series of three steps: (a) fit a model to describe the joint classes; (b) assign membership of the individuals to these classes; (c) fit a regression model of the outcome on the inferred latent class. Schuler et al. (2014) provide a nice discussion of the strengths and weaknesses of each approach. In their conclusions, they suggest that 1-step methods offer many advantages but that one barrier to their implementation is computational. To be precise, estimation based on a joint likelihood for both the latent class and causal effect modeling might have issues with numerical convergence.

Our new contributions to the literature in the current paper are the following:

1. Extension and formalization of the classical potential outcomes framework (Rubin, 1974; Holland, 1986) to accomommodate latent treatment effects. This entails developing the necessary assumptions for definition and identification of an appropriate causal estimate with observed data. This leads to a new quantity, the local latent average causal effect.
2. Development of a new estimation procedure for the local causal effect with latent variables. This leverages techniques from sufficient dimension reduction and partial least squares (Naik and Tsai, 2000).

To our knowledge, the use of latent causal inferential techniques has not been developed for effect estimation in radiomics or genomics. The most related technique comes from genetics, where principal components-type approaches are used to adjust for population stratification, which is a type of confounding (Patterson et al., 2006; Epstein et al., 2007). However, in that setup, latent variables are used to model confounders, not the main effect of interest, which is the focus of the current article. As a proof of concept, we apply our approach to two radiomics datasets in the literature, one from glioblastoma, the other a public available data from osteosarcoma (Zhang et al., 2019).

## 2. Motivating Datasets

In this article, we will use two datasets to illustrate the methodology as a proof of concept. We use these because the distribution of the outcome is different. For the study described in Section 2.1, the outcome is continuous, while for that in Section 2.2, the outcome is binary.

### 2.1. Glioblastoma Multiforme Study

Glioma is the most common type of brain cancer; it develops in the glial cells (Ohgaki, 2009). Among these, glioblastoma multiforme (GBM) is the most frequent and malignant histologic type. Patients with GBM have on average 3% 5-year survival after diagnosis (Ohgaki, 2009). The dataset we work with comes from the Cancer Genome Atlas, consisting of data on 226 subjects with GBM. For these subjects, imaging was done using three protocols: T1, T2 and FLAIR. In this paper, we only focus on the first two. T1 and T2 refer to protocols that utilize two different properties of fMRI. With fMRI, the magnetic current induces a magnetic field, and T1 refers to the speed at which the electron spins in the blood realign with the recovery of the longitudinal orientation. T2 refers to the loss of magnetization as a result of the loss in phase coherence of the electrons. The T1 and T2 images were represented in DICOM format, which was then converted to NIfTI format and processed for standardization with 1mm isovoxel resolution as follows:

1. Postcontrast T1-weighted images (T1C) were resampled to 1mm isovoxel resolution.
2. T2 images were registered to T1C images after skull stripping, using the FMRIB software library (http://fsl.fmrib.ox.ac.uk/fsl/fslwiki/FSL).
3. Image signal intensity was normalized using the WhiteStripe R package.

The tumor areas, defined as areas of T2 hyper-intense tumor and edema on FLAIR images, were segmented by using semi-automatic methods, including signal intensity thresholding, region growing, and edge detection, with an open source software (Medical Image Processing, Analysis and Visualization, https://mipav.cit.nih.gov/). Radiomic features were extracted from all isotropic voxel image segmented regions of interest (ROIs) using pyRadiomics (Van Griethuysen et al., 2017). Extraction settings were configured to features from original images, as well as Wavelet filtered and Laplacian of Gaussian (LoG) filtered images and were calculated considering adjacent voxel in 3 dimensions. In total, 1046 radiomic features were extracted per image.

### 2.2. Osteosarcoma Study

Osteosarcoma is a cancer that usually develops in the cells that form bone, the osteoclasts. It happens most often in children, adolescents, and young adults. In a recent study, Zhang et al. (2021) conducted a study of 102 subjects with osteosarcoma who underwent neoadjuvant chemotherapy. Prior to having treatment, they received a dynamic contrast-enhanced MRI (DCE-MRI) scan. The Response Evaluation Criteria in Solid Tumors were used to evaluate the neoadjuvant chemotherapy response as effective (complete remission and/or partial remission) or ineffective (stable and progressive disease).

Using the Radcloud software platform, Zhang et al. (2021) extracted a total of 1,409 quantitative imaging features. They can be divided into four groups: (a) Group 1 represent typical summaries for the distribution of voxel intensities within the MR image; (b) Group 2 are three-dimensional features that reflect the shape and size of the region; (c) Group 3 are second-order texture features that quantify region heterogeneity differences, calculated from gray-level run length and gray-level co-occurrence texture matrices; (d) Group 4 contains 1,302 first-order statistics and texture features after applying Laplacian, logarithmic, exponential, and wavelet filters on the image. The goal is to see whether or not radiomics can predict treatment response.

## 3. Proposed Methodology

### 3.1. Potential Outcomes Framework

We first review the potential outcomes framework of Rubin (1974) and Holland (1986) and begin by assuming that the treatment is observed. For the sake of exposition, we will assume that it is continuous, similar to Imai and Van Dyk (2004). Let *Z* denote the treatment, with possible values *z*∈*Ƶ*. Define {*Y*_*i*_(*z*):*z*∈*Ƶ*} to the set of potential outcomes for subject *i*, *i* = 1, …, *n*; *Y*_*i*_(*z*) represents the potential outcome for subject *i* with the treatment equals *z*. In addition, we assume the existence of a set of confounders **X**. For proper causal inference within the potential outcomes framework, one needs the assumption based on strong ignorability of the treatment:


(1) $$\{Y(z):z\in Ƶ\}\;\underline{∥}\;Z|X,$$

which in words states that treatment assignment is conditionally independent of the set of potential outcomes given covariates. In the case of binary treatment, Rosenbaum and Rubin (1983) refer to (1) as the strongly ignorable treatment assumption. Heuristically, what (1) implies is that the potential outcomes can be viewed as predefined random variables. The randomness in the populations occurs due to the non-ignorable missing data mechanism, in the sense of Little and Rubin (2019), that makes only one of the potential outcomes observable for each subject. In addition, we make the assumption of consistency so that the observed outcome for a subject coincides with the corresponding potential outcome.

Based on the potential outcomes, we can define the following local causal effect parameter


(2) $$LCE_{i}(z)=Y_{i}(z+1)-Y_{i}(z),\;z\in Ƶ$$

for *i* = 1, …, *n*. We also note the dependence of (2) on the treatment. If we assume that the effect is constant over levels of *Z*, then it would be possible to pool effects to result in a statistically more efficient estimator of the local causal effect.

Two more assumptions that are commonly invoked in the causal inference literature are the positivity assumption and the common support condition. The former states that *E*(*Z*|**X**)≠0 for all possible values of **X**. In other words, there exist no regions of the confounder distribution that preclude observe any possible value of the treatment. The common support condition states that there is sufficient overlap in **X** across all values of *Z*.

If we were to average the local causal effects (2) over all subjects (*i* = 1, …, *n*), then this would correspond to a *local* average causal effect. In the case where the treatment is binary, this effect reduces to the average causal effect that has been considered in the literature.

Analogous to the propensity score of Rosenbaum and Rubin (1983) in the case of binary treatment, we can consider a quantity representing the conditional mean of treatment given confounders, *E*(*Z*|**X**). This is a special case of the generalized propensity score of Imai and Van Dyk (2004) and has several desirable properties. The first is that it reduces the modeling of confounders to modeling a conditional mean of treatment, which reduces the dimension. Second, provided *E*(*Z*|**X**) is correctly modeled, then it functions as a balancing score in that if (1) holds, then {*Y*(*z*):*z*∈*Ƶ*}||*Z*|*E*(*Z*|**X**). The properties of *E*(*Z*|**X**) lead to a natural strategy for performing causal inference:

1. Fit a model for *E*(*Z*|**X**).
2. Given the predicted conditional mean in (1), perform a regression of *Y* on *Z* in which an adjustment is made using *E*(*Z*|**X**).

There is a variety of approaches to perform adjustment in the second step of this algorithm. These include inverse weighting methods, regression adjustment, matching, subclassification, or a combination thereof. Please see Lunceford and Davidian, 2004 for more discussion on these methods.

### 3.2. Latent Treatment: Model Formulation and Estimation

We now relax the assumption that the treatment *Z* be observed. Instead, we now have available several observations **U**≡(*U*_1_, …, *U*_*K*_) that capture the latent treatment variable *Z*. A commonly used assumption here is the so-called local independence assumption (Henning, 1989), which states that conditional on *Z*, *U*_1_, …, *U*_*K*_ are conditionally independent. We can then factorize the joint distribution of the potential outcomes (denoted here as *Y*(·)) *Z*, **X** and **U** as


$$\begin{array}{l} f_{Y(\cdot ),Z,X,U}=f_{Y(\cdot )|Z,X,U}f_{Z|X,U}f_{X,U}\\ \;=f_{Y(\cdot )|U,X}f_{Z|X,U}f_{X,U}, \end{array}$$

where we have the assumption (1) to simplify the conditional distribution going from the first line to the second. If we further assumption that the joint density *f*_**X**, **U**_ are ancillary for the causal parameters of interest, then we can base our approach to inference by specifying likelihoods corresponding to *f*_*Y*(·)|**U**, **X**_ and *f*_*Z*|**X**, **U**_, respectively. Recapping, here are the assumptions we need for valid causal effect estimation of the LCE in the latent case:

1. *Z*||{*Y*(*z*):*z*∈*Ƶ*}.
2. The components of **U** are conditionally independent given Z.
3. The components of **U** are conditionally independent of any variable *S* given Z.
4. *E*(*Z*|**X**) exists for all **X**.

We note that Assumptions 2 and 3 look similar but are conceptually different. The former deals with the radiomics features providing conditionally independent information given the tumor and is referred to as local independence. By contrast, the latter has to do with measurement invariance (Meredith, 1993), namely that the radiomics measurements are capturing the same concept independent of other variables. In fact, it is assumption 3 that is a very important one if one wishes to have any chance of the radiomics data analyses reflecting potentially generalizable findings.

**Figure 1:**
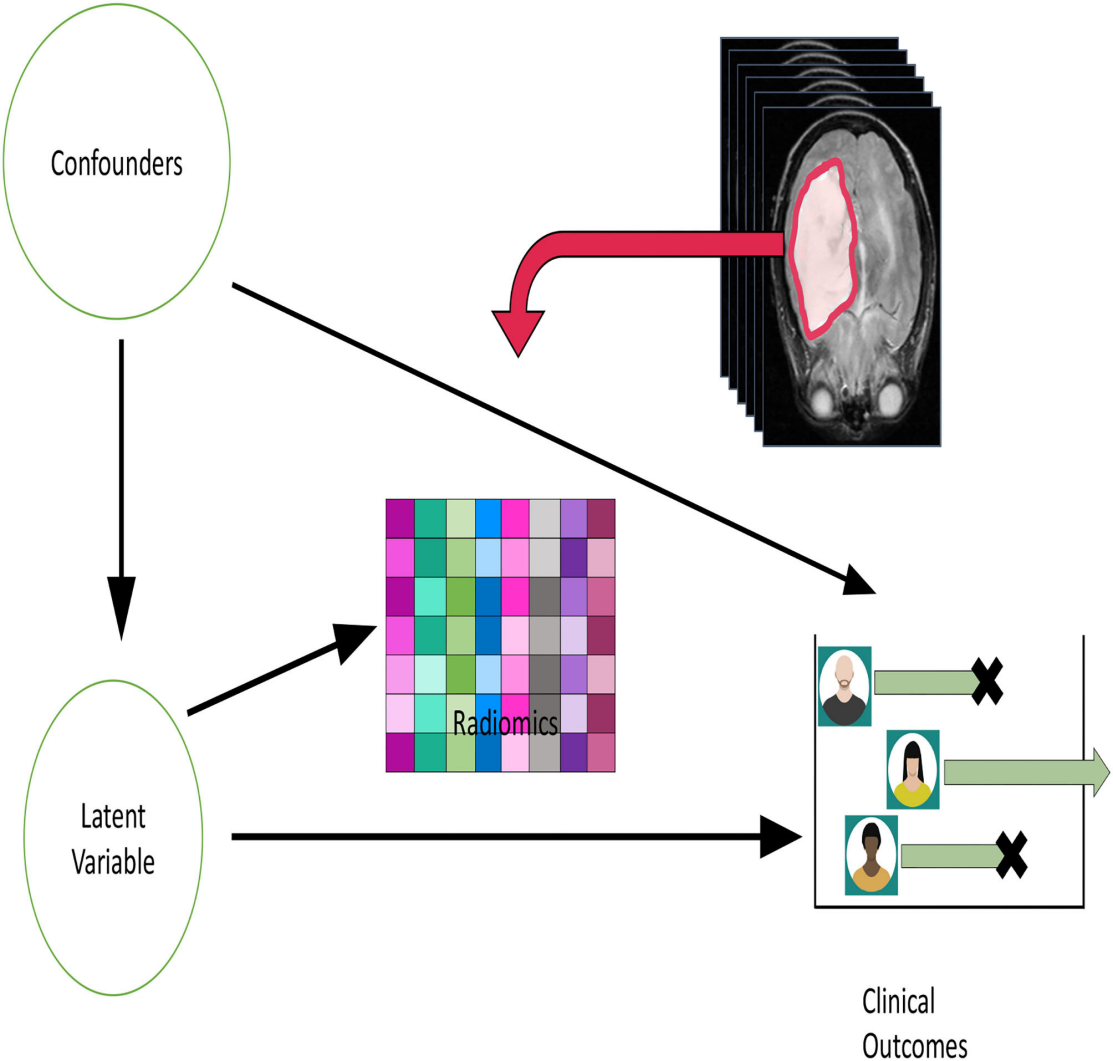
A conceptual model diagram relating confounders, radiomics and outcome variables in medical studies. The goal is to estimate the causal effect corresponding from the arrow from the “Latent variable” circle to the clinical outcome.

Figure 1 depicts our conceptual model. We could then convert Figure 1 into a causal diagram (Greenland et al., 1999) which leverages the graphical model for causation (Pearl, 2009). We can use the traditional rules about directed acyclic graphs to model conditional independence. In particular, the observed radiomics data will be conditionally independent given the latent variable. In addition, the latent variable d-separates (Pearl, 2009) the confounders from **U**. The causal effect we focus on in Figure 1 is that from the latent variable to the clinical outcome. This separation of the scientific estimants (i.e., causal effects) from the data represent one of the appealing features of Figure 1.

We note that the structure of Figure 1 is related to diagrams used by practitioners of structural equations modeling (SEM, Bollen and Pearl, 2013). In that literature, relationships between latent variables are referred to as the structural model, while those relating latent to measured are called measurement models. SEM combines the two types of models in order to induce a joint distribution for the observed data which is then used for estimation and inference. As described by Bollen and Pearl (2013), the structural model is consistent with the potential outcomes framework that we outlined in Section 3.1.

Thinking of the radiomics data as the main effect in a causal analysis is consistent with a tumor progression model in which the cancer's behavior at clinical diagnosis matters, and any biological preceding events can only play the role of confounders. Biologically, this means that confounders increase the propensity for tumorigenesis to occur.

## 4. Methodology

### 4.1. Proposition and Partial Least Squares

Based on the assumptions and conditional independence statements we have laid out in the previous sections, we have following proposition.

**Proposition 1**. The random variable *Z* d-separates both **U** and **X** and *Y* and **X**.

While proposition 1 is quite simple in nature, it in fact reveals a powerful result and leads to a new approach to causal effect estimation. In particular, we can estimate *Z* as a latent variable in two simultaneous regression models: (1) **U** on **X**; (2) *Y* on **X**. This simultaneous estimation has potential benefits largely due to the absence of direct arrows from **U** to *Y* in Figure 1. We have thus converted a causal effect estimation problem into a multi-task learning problem with a latent variable.

Our algorithms for causal effect estimation will be based on partial least squares (PLS) (Helland, 1988, 1990). PLS presumes that **U**_*i*_ and **X**_*i*_ (*i* = 1, …, *n*) are both linear functions of a set of common latent factors. An alternative characterization for PLS was given by Stone and Brooks (1990). Suppose that we are fitting the following model:


(3) $$E(Y_{i}|Z_{i})=Z_{i}^{T}\beta_{0}$$

Then, Stone and Brooks (1990) consider the following class of objective functions:


$$\text{Var}{\left(Z_{i}^{T}\beta \right)}^{2}\text{Cov}{\left(Y_{i},Z_{i}^{T}\beta \right)}^{\alpha /(1-\alpha )-1},$$

where Var and Cov and short-hand notation for variance and covariance, and α is a number between 0 and 1. In this framework, values of α = 0, α = 1/2 and α = 1 correspond to the objective functions maximized by ordinary least squares (OLS), partial least squares (PLS) and principal components regression (PCR), respectively. Using this framework, we find that the PLS algorithm acts as some hybrid between the usual least squares estimator and the principal components regression approach of Massy (1965).

The algorithm for multivariate PLS that we will use is the kernel algorithm proposed by Dayal and MacGregor (1997). We assume that the columns of **U** and **X** are centered and scaled. Recall again that the PLS model formulation is given by


(4) $$\left(\begin{array}{c} X=TP^{\prime }+E\\ U=VQ^{\prime }+F \end{array}\right),$$

where **T** and **V** are *n* × *l* matrices, and **P** and **Q** are the so-called locaing matrices corresponding to **X** and **U**, respectively. The dimension of **P** is *p* × *l*, while for **Q**, it is *m* × *l*. The matrices **E** and **F** are the error terms with entries being independent and identically distributed normal random variables with mean zero and variance σ^2^.

The kernel algorithm proceeds as follows:

1. Compute the matrices **X**′**X** and **X**′**U**.
2. Let *b* = 1. Compute **q**_1_ as the eigenvector corresponding to the largest eigenvalue of **U**′**X**′**XU**. Then set $w^{\prime }{}_{b}={\left(X^{\prime }U\right)}_{b}q_{b}$ and rescale the entries of **w**_*b*_ to have unit norm. For *b* = 1, ${\left(X^{\prime }U\right)}_{b}=X^{\prime }U$; for *b* > 1, its definition will be given in (5).
3. Compute **r**_*b*_. For *b* = 1, **r**_1_ = **w**_1_, while for *b* > 1,

$$r_{b}=w_{b}-\sum_{a=1}^{t-1}p_{a^{\prime }}w_{b}r_{b}.$$

4. Compute **t**_*b*_ = **Xr**_*b*_, $p_{b}=t^{'}{}_{b}X/t^{'}{}_{b}t_{b}$ and $q^{\prime }{}_{b}=r^{\prime }{}_{b}(X^{\prime }U)/t^{\prime }{}_{b}t_{b}$.
5. Compute

(5) $${\left(X^{\prime }U\right)}_{b+1}={\left(X^{\prime }U\right)}_{b}-p_{b}q^{\prime }{}_{b}(t^{\prime }{}_{b}t_{b})$$

1. Repeat steps (2)-(5).

At the end, the regression coefficients are given by the outer product of the matrix consisting of **r**_*b*_ and that consisting of **q**_*b*_. This kernel algorithm has been implemented in the kernelpls.fit function that is available in the pls package (Wehrens and Mevik, 2007).

### 4.2. Theoretical Justification

Partial least squares methods were given a justification for the single-index model by Naik and Tsai (2000). This was done by incorporating ideas from the field of sufficient dimension reduction (Li, 2018). This is a branch of statistics in which the goal is to develop “model-free” procedures in order to summarize data while preserving regression relationships. The field started with the observation by Brillinger (2012) in which ordinary least squares methods provide estimates that were consistent up to a sign for regression parameters in more general single-index models. A recent overview of the field can be found in Li (2018).

We now develop a multivariate extension of the results of Naik and Tsai (2000). To do this, we consider a multivariate response for each subject that can be summarized as a an *K*−dimensional vector **U** along with a *p*−dimensional vector of covariates **X**.

We then formulate the following multivariate regression model:


(6) $$\left(\begin{array}{c} U_{1}\\ \vdots \\ U_{K} \end{array}\right)=\left(\begin{array}{c} g_{1}(\beta_{1^{\prime }}X,\epsilon_{1})\\ \vdots \\ g_{K}(\beta_{K^{\prime }}X,\epsilon_{K}), \end{array}\right)$$

where *g*_*j*_ (*j* = 1, …, *K*) are monotonic functions in both arguments and ϵ_1_, …, ϵ_*K*_ are random vectors representing the error distributions for the models. In (6), the *p*−dimensional parameter vectors β_1_, …, β_*K*_ specify the directions of interest. In the case where *K* = 1, model (6) reduces to a generalized single-index model. We make the following assumptions.

*Assumption 1*. **X** has a multivariate normal distribution. *Assumption 2*. The covariance matrices *n*^−1^**X**′**X** and *n*^−1^**X**′**U** converge in probability to limits Σ_*xx*_ and Σ_*xu*_, respectively. *Assumption 3*. Taken as an operator, the range of Σ_*xx*_ coincides with the range of Σ_*xu*_.

Based on these three assumptions, we have the following result.

**Theorem:** Under Assumptions 1–3, the multivariate PLS estimator converges in probability to a constant times (β_1_, …, β_*m*_).

**Proof:** The multivariate PLS estimator can be expressed as $\hat{R}{\left({\hat{R}}^{\prime }n^{-1}X^{\prime }X\hat{R}\right)}^{-1}{\hat{R}}^{\prime }n^{-1}X^{\prime }U$, where $\hat{R}$ is the matrix derived from the Kryvlov sequence of matrices of *n*^−1^**X**′**X** and *n*^−1^**X**′**U**. By assumption 2, this will converge to $R{\left(R^{\prime }\Sigma_{xx}R\right)}^{-1}R^{\prime }\Sigma_{xu}\beta^{*}$. By assumption 3, β^*^ will the in the space spanned by *R* so that $\Sigma_{xx}^{1/2}\beta^{*}$ will be in the space spanned by $\Sigma_{xx}^{1/2}R$. The statement of the theorem then follows.

One of the major assumptions in traditional sufficient dimension reduction procedures has been the linearity assumption, which is satisfied by multivariate normal distributions and more generally, elliptically symmetric distributions. This gets violated in situations with discrete predictors. Recently, Ghosh (2022) has developed an interpretation of sufficient dimension reduction methods from an information-theoretic point of view. In this interpretation, the partial least squares algorithm can be viewed as an information-maximization operation under less restrictive distributional assumptions than those required in Theorem 1 of the paper.

### 4.3. Integration With Causal Modeling and Implementation Details

In examining Figure 1, we propose the following strategy for causal effect estimation.

1. Run a multivariate PLS regression of the radiomics data on the confounders.
2. Using the scores as the inferred latent treatment from the output of the PLS regression, perform causal inference of the treatment on outcome.

We have lots of choices on how to perform step 2. above. Our approach is to use regression adjustment as a means of inferring causal effects. For the standard error, we will use the non-parametric bootstrap (Efron, 1981).

We note that the outcome of the multivariate PLS can be fairly general. These include variables that are continuous, binary or unordered categorical. For right-censored failure time variables, we use the suggestion of Keleş and Segal (2002) and compute a first-stage martingale residual from a null model (i.e., one with no covariates). We then treat the residual as a continuous variable to be input into the partial least squares algorithm.

## 5. Numerical Examples

### 5.1. Glioblastoma Multiforme Study

**Figure 2:**
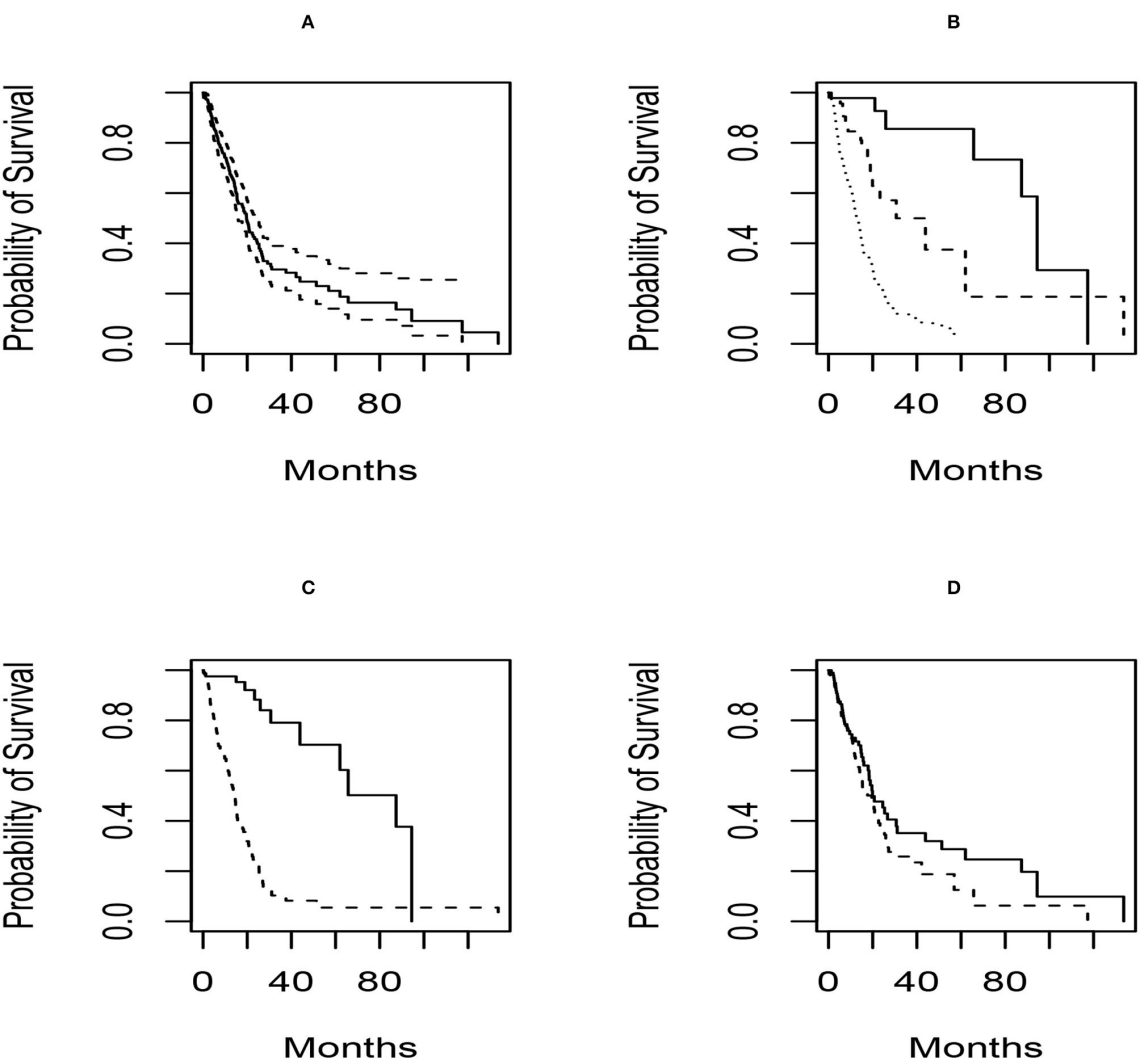
Survival distribution plots for the subjects in the glioblastoma radiomics study. For all plots, the x axis represents the time in weeks and the y-axis is the survival probability. Panel **(A)** shows the Kaplan–Meier plot for the entire population, along with associated 95% pointwise confidence intervals. Panel **(B)** shows the Kaplan–Meier estimates by grade (solid = grade 2; dashed = grade 3; dotted = grade 4). In Panel **(C)**, the survival distributions by IDH mutation status (solid = mutant; dashed = wild-type) are presented. Finally, the gender-specific survival distributions (solid = female; dashed = male) are given in **(D)**.

The confounders available in this analysis are gender, grade, and IDH mutation status. Mutations of the isocitrate dehydrogenase (IDH1) gene has been shown to be a marker of oncogenesis and is one of the most specific biomarkers in the diagnostic classification of secondary GBM (Dang et al., 2010) Gliomas with mutated IDH have improved prognosis compared to gliomas with wild-type IDH and are detected by immunohistochemistry and magnetic resonance (MR) spectroscopy (Cohen et al., 2013). We first characterize survival in the population and by grade, IDH mutation status and gender. These are presented in Figure 2.

Based on the plots, we see that there are differences in survival based on grade and IDH mutation status and not for gender. We also note that 22 subjects are missing IDH mutation, while 1 is missing grade and gender.

We next applied the latent causal effect approach in the paper with two sets of confounders: (gender, grade) and (gender, grade, IDH mutation statu). We note that the former analysis will have 225 subjects, while the latter will have 203. The results are shown in Table 1.

**Table 1:** Latent causal effects and associated confidence intervals in glioblastoma study.

**Confounders**	***n***	**Estimate**	**95% confidence interval**
Gender, grade	225	−0.19	(−0.27, −0.08)
Gender, grade, IDH	203	−0.20	(−0.26, −0.11)

*The estimates denote the average latent local causal effect of radiomics on survival. There are two analyses reported here. The first row denotes an analysis in which gender and grade are confounders. The second row represents an analysis in which gender, grade, and IDH mutation status are confounders. The n refers to the sample size used. The estimate is the estimated causal effect using the methods in Section 4. The 95% confidence intervals are obtained using the non-parametric bootstrap*.

The model that is being fit is for the adjusted survival time as a function of the latent variable. Based on the analysis, we see that there is a highly significant effect of the treatment on outcome. Both analyses that higher values of the latent construct are associated with lower adjusted times to death.

### 5.2. Osteosarcoma Study

In this dataset, there is only one confounder, the stage of cancer (stage IIB vs. not). Of the 102 subjects, 75 were stage IIB. The effect of radiomics on treatment response was analyzed here. The multivariate PLS algorithm yields an average causal effect of 0.099, with an associated 95% confidence interval of (−0.13, 0.20). As an alternative, we computed the first principal components using the radiomics data and fit a regression model of treatment response *Y* on the principal component and stage. Based on the fitted model, we obtained the following equation:


Y=0.29-0.02PC1+0.23Stage,

with associated standard errors of 0.01 and 0.11 for the principal component and stage. While both variables are statistically significant at a 0.05 level of significance (p-values of 0.05 and 0.03 for PC score and stage, respectively), we note that the direction of the effect is reverse that from the multivariate PLS approach.

## 6. Discussion

In this article, we have introduced an approach to causal modeling with radiomics data. The assumptions needed for identification of the causal effects from observed data along with an estimation procedure for causal effects. We have demonstrated the application of the methodology to two radiomics datasets in cancer. Further evaluations are need to demonstrate validation of the methodology. To help readers, we have made code available to do the analysis with the osteosarcoma data at the following location: http://github.com/GhoshLab/CausalRadiomics.

**Figure 3:**
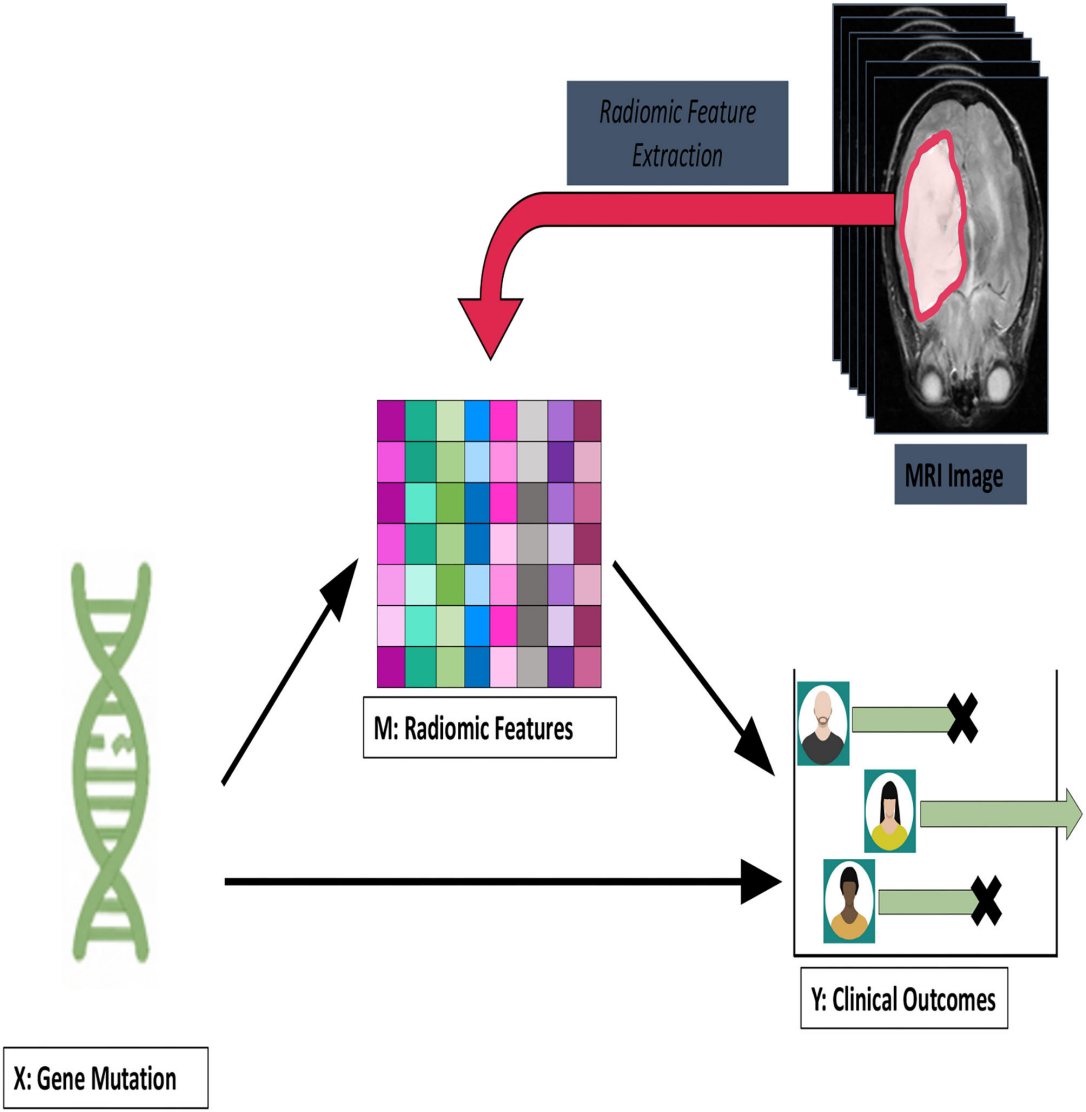
A framework for viewing the radiomics measurements as mediation variables. The exposure here is a DNA mutation which leads to tumorigenesis that is captured by the imaging and radiomics feature and which leads to a clinical outcome.

An alternative approach to radiomics data is to treat it as a mediation variable in a causal effects analysis. There, it would be an intermediate variable, and genetic data would serve as the main exposure. Viewing the radiomics as mediation variable makes explicit the role of initiating events. In Figure 3, we suggest a DNA mutation as the beginning event, but other choices could be entertained.

In this setup, the radiomics is viewed as a downstream event, and the mutation will have effects on survival as mediated through the radiomics and effects outside the pathway. In Huang and Pan (2016), the authors used microRNA as the exposure and gene expression from several pathways as the mediators. For this setup, they develop tests of mediation and associated testing procedures. More recently, in Aung et al. (2020), a Bayesian approach to mediation analysis as developed, in which methylation profiles were the high-dimensional mediators of a univariate exposure. Their algorithm was based on a variable selection procedure with shrinkage priors to select for mediators. Finally, we note the principal mediation directions approach of Chén et al. (2018). For their application, subject-level functional magnetic resonance imaging profiles were the mediator, and the goal was to understand the areas of the brain that mediated pain-invoked stimuli. In Chén et al. (2018), the authors used a supervised principal components approach similar to what is presented here. We will explore extensions of our latent causal inference procedures to the mediation problem in future work.

## Data Availability Statement

The data are available from YSC upon reasonable request. Requests to access these datasets should be directed to yoonseong.choi07@gmail.com.

## Ethics Statement

Ethical review and approval was not required for the study on human participants in accordance with the local legislation and institutional requirements. Written informed consent for participation was not required for this study in accordance with the national legislation and the institutional requirements.

## Author Contributions

DG led the conceptualization of the project, methodological development, analysis, and writing of the first draft of the manuscript. All authors participated in the writing process and approved the final manuscript.

## Funding

EM was supported by T15 LM009451.

## Conflict of Interest

The authors declare that the research was conducted in the absence of any commercial or financial relationships that could be construed as a potential conflict of interest.

## Publisher's Note

All claims expressed in this article are solely those of the authors and do not necessarily represent those of their affiliated organizations, or those of the publisher, the editors and the reviewers. Any product that may be evaluated in this article, or claim that may be made by its manufacturer, is not guaranteed or endorsed by the publisher.
